# Occurrence data for the two cryptic species of *Cacopsylla pruni* (Hemiptera: Psylloidea)

**DOI:** 10.3897/BDJ.9.e68860

**Published:** 2021-07-01

**Authors:** Nicolas Sauvion, Jean Peccoud, Christine N Meynard, David Ouvrard

**Affiliations:** 1 National Research Institute for Agriculture, Food and Environment (INRAE), Montpellier, France National Research Institute for Agriculture, Food and Environment (INRAE) Montpellier France; 2 PHIM, Univ Montpellier, INRAE, CIRAD, Montpellier SupAgro, Montpellier, France PHIM, Univ Montpellier, INRAE, CIRAD, Montpellier SupAgro Montpellier France; 3 UMR CNRS 7267 Ecologie et Biologie des Interactions, Equipe Ecologie Evolution Symbiose, Université de Poitiers, Poitiers, France UMR CNRS 7267 Ecologie et Biologie des Interactions, Equipe Ecologie Evolution Symbiose, Université de Poitiers Poitiers France; 4 CBGP, INRAE, CIRAD, IRD, Montpellier SupAgro, Univ Montpellier, Montpellier, France CBGP, INRAE, CIRAD, IRD, Montpellier SupAgro, Univ Montpellier Montpellier France; 5 ANSES-Laboratoire de la Santé des Végétaux, Montpellier, France ANSES-Laboratoire de la Santé des Végétaux Montpellier France

**Keywords:** Hemiptera, psyllid, *Cacopsylla pruni*, vector-borne plant pathogen, phytoplasma, '*Candidatus* phytoplasma prunorum', European stone fruit yellows, species distribution, epidemiology

## Abstract

**Background:**

*Cacopsylla pruni* is a psyllid that has been known since 1998 as the vector of the bacterium ‘*Candidatus* Phytoplasma prunorum’, responsible for the European stone fruit yellows (ESFY), a disease that affects species of *Prunus*. This disease is one of the major limiting factors for the production of stone fruits, most notably apricot (*Prunus armeniaca*) and Japanese plum (*P. salicina*), in all EU stone fruit-growing areas. The psyllid vector is widespread in the Western Palearctic and evidence for the presence of the phytoplasma that it transmits to species of *Prunus* has been found in 15 of the 27 EU countries.

Recent studies showed that *C. pruni* is actually composed of two cryptic species that can be differentiated by molecular markers. A literature review on the distribution of *C. pruni* was published in 2012, but it only provided presence or absence information at the country level and without distinction between the two cryptic species.

Since 2012, numerous new records of the vector in several European countries have been published. We ourselves have acquired a large amount of data from sampling in France and other European countries. We have also carried out a thorough systematic literature review to find additional records, including all the original sources mentioning *C. pruni* (or its synonyms) since the first description by Scopoli in 1763. Our aim was to create an exhaustive georeferenced occurrence catalogue, in particular in countries that are occasionally mentioned in literature with little detail. Finally, for countries that seem suitable for the proliferation of *C. pruni* (USA, Canada, Japan, China etc.), we dug deeper into literature and reliable sources (e.g. published checklists) to better substantiate its current absence from those regions.

Information on the distribution ranges of these vector psyllids is of crucial interest in order to best predict the vulnerability of stone fruit producing countries to the ESFY threat in the foreseeable future.

**New information:**

We give free access to a unique file of 1975 records of all occurrence data in our possession concerning *C. pruni*, that we have gathered through more than twenty years of sampling efforts in Europe or through intensive text mining.

We have made every effort to retrieve the source information for the records extracted from literature (1201 records). Thus, we always give the title of the original reference, together with the page(s) citing *C. pruni* and, if possible, the year of sampling. To make the results of this survey publicly available, we give a URL to access the literature sources. In most cases, this link allows free downloads of a PDF file.

We also give access to information extracted from GBIF (162 exploitable data points on 245 occurrences found in the database), which we thoroughly checked and often supplemented to make the information more easily exploitable.

We give access to our own unpublished georeferenced and genotyped records from 612 samples taken over the last 20 years in several European countries (Switzerland, Belgium, Netherlands, Spain etc.). These include two countries (Portugal and North Macedonia), for which the presence of *C. pruni* had not been reported before. As our specimens have been genotyped (74 sites with species A solely, 202 with species B solely and 310 with species A+B), our new data enable a better overview of the geographical distribution of the two cryptic species at the Palaearctic scale.

## Introduction

Psyllids (Psylloidea), or jumping plant-lice, are plant sap-sucking hemipterans that could be considered as a minor group in terms of species diversity (3,573 described species according to Ouvrard 2021, compared to 104,165 hemipteran species according to Zhang 2013). However, a few psyllids are amongst the most devastating pests of annual and perennial crops due to their ability to transmit phytopathogenic bacteria causing significant agricultural losses. For example, *Bactericera cockerelli* (Šulc, 1909) is the vector of a liberibacter responsible for Zebra chips (ZC), a disease that caused millions of dollars in losses to the potato industry in the United States, Mexico, Central America and New Zealand, often leading to the abandonment of entire fields (Munyaneza 2012). The huanglongbing (HLB), the world's most devastating disease of trees of species of *Citrus*, is associated with two psyllid species, *Diaphorina citri* (Kuwayama, 1908) and *Trioza erytreae* (Del Guercio, 1918) (Bové 2006, Gottwald 2010, Khamis et al. 2017, Shimwela et al. 2016, Rwomushana et al. 2017, Ajene et al. 2020). In 2014, *T. erytreae* was fortuitously discovered in Spain and Portugal in parks and avenues and even in privately owned trees during a survey for other citrus pests (Arenas-Arenas et al. 2018). Although circum-Mediterranean species of *Citrus* have been, thus far, spared from the disease, the sporadic records of *T. erytreae* in these regions exposes them to the threat of a potential devastating epidemic (Cocuzza et al. 2017).

Other bacteria transmitted by psyllids to fruit trees have major economic impacts, in Europe in particular (Hadidi et al. 2011). These are phytoplasmas of trees of species of *Prunu*s, as well as apple and pear trees, transmitted by psyllids of the genus *Cacopsylla*. These, respectively, cause the European stone fruit yellows (ESFY), the Apple Proliferation (AP) and the Pear Decline (PD) (Jarausch et al. 2019). These bacteria and their vectors are native to Europe where they occur widely in orchard as well as wild habitats, preventing the eradication of the vectors and, therefore, containment of the diseases. The psyllid vectors are controlled mainly by insecticides, but the evolution of farming practices (e.g. reduction in the use of pesticides) and European regulations (i.e. pathogens removed from the list of quarantine organisms) could be the source of new emergences in the near future. In spite of great efforts from the European research community to better understand the biology and the ecology of the psyllid vectors of phytoplasmas (COST Action FA0807 2013, MacLeod et al. 2012), the presence of these insects in some parts of Europe and even in other parts of the world impacted by these diseases, remains unclear (Steffek et al. 2012). Resolving this uncertainty would help to assess the risks posed by the fruit tree phytoplasmas (MacLeod et al. 2012) and to make decisions to manage these risks.

Dispersal of psyllid vectors poses a threat to food security across countries, stressing the need to anticipate the risks associated with introductions of new psyllids. Mapping the vector potential distributions under scenarios of introduction is crucial to an efficient pest risk assessment (PRA) framework (Venette et al. 2010). Occurrences representing the extent and variability within the current range of a given species are key to characterise and map its potential distribution under scenarios of introduction or climate change. Species distribution models (SDMs) have become the main predictive tool to achieve this goal (Elith and Leathwick 2009, Guisan et al. 2013). SDMs have proven their usefulness, inter alia, in invasion biology (e.g. Meynard et al. 2017, Syfert et al. 2017) and in conservation biology (e.g. Guisan et al. 2013, Muscatello et al. 2021). In plant pathology, SDMs are also increasingly used to predict the potential distributions of vector-borne plant pathogens (e.g. Benhadi-Marn et al. 2020, Narouei-Khandan et al. 2016, Shimwela et al. 2016). However, the reliability of these models heavily depends on the quality of the occurrence data that are used as input to map species distributions.

At least four criteria should be considered before using occurrence data as input for SDMs (Meynard et al. 2019): geographic and environmental representation and extent, quantity, accuracy of the georeferenced records and accuracy of the taxonomic identification. In short, occurrence data points should represent the full extent of biodiversity within the environments that the species is able to occupy, they should be numerous enough to allow its characterisation and geographic coordinates and taxonomic identification should be accurate, as these may otherwise introduce error in the modelled occurrence-environment correlations. High-quality data to properly map a species’ distribution are often difficult to obtain, especially in insects. Indeed, collecting insects and information on their biology is often a time-consuming process that requires high taxonomic expertise. Insect species identification may necessitate painstaking morphological analyses or even the development of specific tools such as molecular markers (e.g. Peccoud et al. 2013). Recent studies have shown that different populations or genotypes within the same taxon can represent different risks, resulting in strikingly different SDM outputs (Meynard et al. 2017, Chardon et al. 2020). Genotypic information throughout the species range can therefore be crucial in the risk assessment process.

Historical data may also consitute a precious resource to help trace vector dispersion routes or simply to access specimens that can no longer be obtained (e.g. samples from an inaccessible locality). Many museums and academic institutions hold field notebooks and maintain collections that are a rich source of valuable information (e.g. collection date and locality) on insect specimens collected during scientific expeditions (Graham et al. 2004, Lister and Climate Change Research Group 2011, Suarez and Tsutsui 2004). Such data have proven useful in reconstructing the history of human or animal infectious diseases and in identifying their sources or reservoirs, in particular for mosquito-borne pathogens (e.g. West Nile virus, Suarez and Tsutsui 2004). To our knowledge, however, this task has never been undertaken for vector-borne plant diseases and historical records appear underexploited, even if they concern regions where such diseases have been endemic for tens to hundreds of years.

Cacopsylla (Thamnopsylla) pruni (Scopoli, 1763) has been known since 1998 as the vector of a bacterium, ‘*Candidatus* Phytoplasma prunorum’ responsible for ESFY (Carraro et al. 1998) and is currently listed as a Regulated Non-Quarantine Pest (RNQP) in Annex IV-Part D of the European Council Directive 2019/2072 (EUR-Lex 2020). This psyllid is widespread in the Western Palearctic (Ouvrard 2021) and the phytoplasma it transmits are reported in 15 of the 27 EU countries (Steffek et al. 2012). ESFY is one of the major factors limiting the production of stone fruits, most notably apricot (*Prunus armeniaca* L.) and Japanese plum (*Prunus salicina* Lindl.) in all EU stone fruit-growing areas. These areas include the three most important apricot producing countries, Spain, Italy and France, which provided 73% of the EU apricot production in 2012 according to Eurostats. In the last twenty years, great efforts have been made to characterise the biology of the ESFY vector (Peccoud et al. 2013, Peccoud et al. 2018), the life cycle of the transmission (Thébaud et al. 2009), the genetic variability of the pathogen (Danet et al. 2011, Marie-Jeanne et al. 2020) and the risk factors of the disease (Marie-Jeanne et al. 2020, Thébaud et al. 2006). However, despite these efforts and the rigorous sanitary control of fruit trees as part of the certification process, the disease continues to pose great problems to fruit growers in Europe, which raises the question of the origin of contaminations in orchards.

In their review, Steffek et al. (2012) pointed out important uncertainties that could undermine the management of ESFY. The rate of psyllid dispersal at various scales (i.e. a growing region, country, Europe or even larger), by natural means or human transportation and the risk of introduction and establishment in new countries were two of the essential issues that remained unresolved. The presence of the vector in several countries from the southernmost part of Europe (Portugal, southern Spain, Greece etc.) which can be directly impacted by ESFY, as well as neighbouring countries, remains undetermined. At the time of the Steffek et al. (2012) review, preliminary studies had shown that *C. pruni* was composed of two genetic groups, then called "biotypes" (Sauvion et al. 2007, Sauvion et al. 2009). However, no detailed data were available on the European distribution of these two biotypes, which were analysed jointly in the Steffek et al. review.

**Figure 1.**
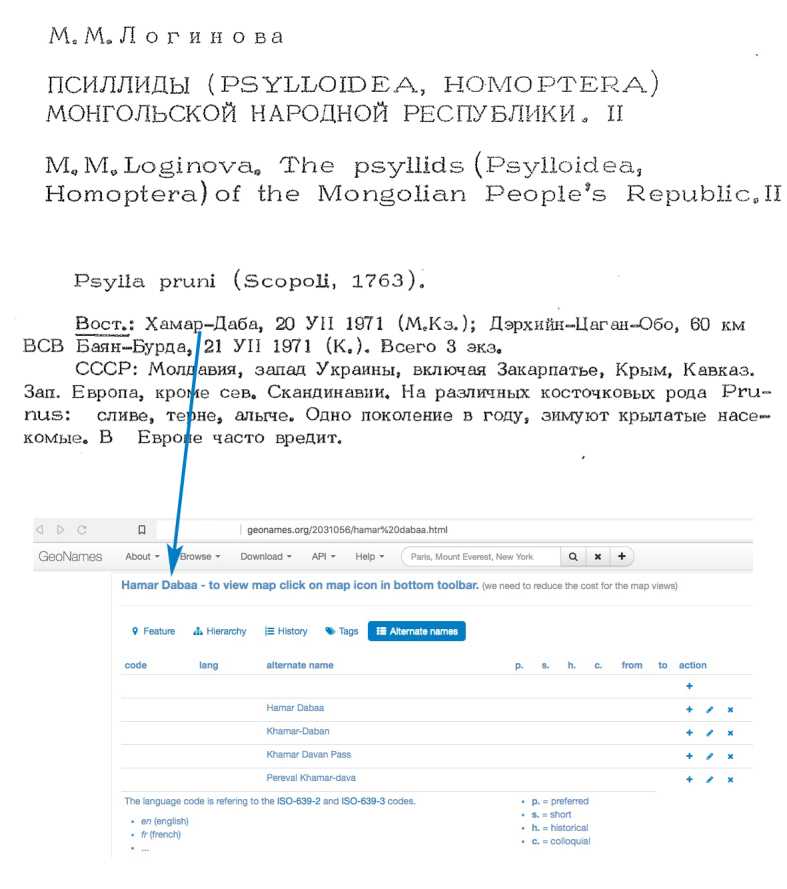
Excerpt from a 1974 article from Loginova referring to *Cacopsylla pruni*, with translation and information about one of the localities cited, Hamar data. After Loginova (1974).

Establishing the geographic distribution of *C. pruni* and possibly for each biotype, was therefore a priority. To this end, we developed molecular markers to easily identify the *C. pruni* biotypes (Peccoud et al. 2013), which allowed us to establish their species status (Peccoud et al. 2018). Numerous new surveys on the presence of *C. pruni* in several European countries have been published (e.g. Etropolska et al. 2015, Jarausch et al. 2019, Sabaté et al. 2016, Seljak 2020, Warabieda et al. 2018), sometimes with a distinction between the two species. In our own laboratory at INRAE-Montpellier, we obtained a large collection of samples through twenty years of surveys in France and other European countries (Portugal, Spain, Belgium, Switzerland, Italy etc.). Some of these samples have been used in publications, but the vast majority have not yet been released in a georeferenced format. We were also able to find unpublished and valuable information in GBIF (e.g. metadata from Natural History Museum of London). Recently, we conducted an extensive literature survey for the original sources mentioning *C. pruni*, as a mean of verification, but more importantly, to precisely locate the source of each specimen. This laborious work often resembled a treasure hunt with its typical pitfalls and puzzles, such as correctly translating Mongolian locality names from a text written in Russian and then georeferencing them (Fig. 1). Sparing others these obstacles was part of our motivation to make the results of this survey publicly available.

Our objective is to give access, through a unique dataset, to all the data we have gathered on the two cryptic species of *C. pruni*. In this way, we hope to contribute to a better management of ESFY in countries affected by the disease and to a better anticipation of the risk of introduction in countries not yet affected.

## General description

### Purpose

**Figure 2.**
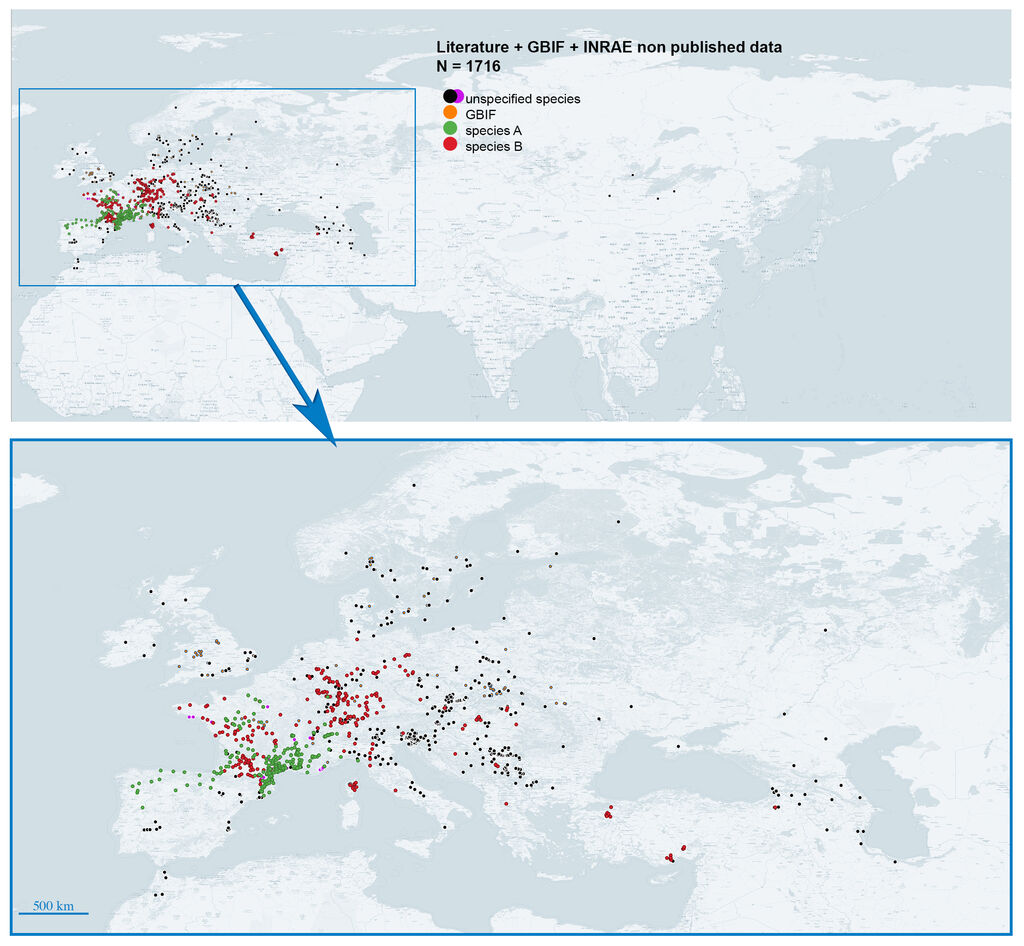
Global map of the 1716 occurrence data available in the *C. pruni* dataset (map generated with QGIS 3.14). The map shows the distribution of cryptic species A (green dots) and B (red dots) according to available data. However, most of the data from the literature (black dots), GBIF (orange dots) or the Psylloidea catalogue of the "Faune de France" (currently being published) do not allow a distinction to be made between cryptic species.

This dataset is a compilation that is meant to include all available information (literature, GBIF, INRAE unpublished data) on the geographical distribution of two cryptic species of the psyllid *Cacopsylla pruni* at the scale of the Palaearctic (Fig. 2). We aimed to publish third-party data that can be otherwise hard to access and first-party data that are not yet published and to ensure free, open access to that information.

## Sampling methods

### Study extent

The data contained in this dataset have three different origins: a systematic literature review, the Global Biodiversity Information Facility [GBIF] network and field collections by researchers/students from INRAE-Montpellier. They cover several ecoregions of the Palaearctic (Fig. 2): the Euro-Siberian region, the Mediterranean Basin, the Western and East Asia (Northern parts). No data were found for Central Asia nor for the Nearctic, despite the known presence of trees of species of *Prunus* and conifers on which *C. pruni* could make its life cycle.

### Sampling description

**Literature data**

In order to extend upon the Steffek et al. (2012) review, we have undertaken a new systematic literature survey for articles/manuscripts/books using the keyword "*Cacopsylla pruni*", its previous combinations "*Chermes pruni" and "Psyllapruni*" or its synonym "*Psylla fumipennis*". To this end, we used the Google Scholar search engine (https://scholar.google.com/) and we explored several scientific databases (AGRICOLA, Agris, CAB Abstract, Web of Science), as well as other types of databases more or less specialised on the subject:

- Psyl’list (https://www.hemiptera-databases.org/psyllist/), an online database dedicated to jumping plant lice;
- National Inventory of Natural Heritage (https://inpn.mnhn.fr/accueil/index), the French portal for biodiversity and geodiversity;
- ISTEX (https://www.inist.fr/services/acceder/istex/) a platform offering the French higher education and research community access to more than 23 million articles from all scientific disciplines and which cover a very long period (from ~ 1400 to 2019);
- Collections of the Natural History Museum of London (https://www.nhm.ac.uk/our-science/collections.html);
- Gallica (https://gallica.bnf.fr), the digital library of the BNF (Bibliothèque Nationale de France);
- Biodiversity Heritage Library [BHL] (https://www.biodiversitylibrary.org/), the world’s largest open access digital library for biodiversity literature and archives.

**Figure 3.**
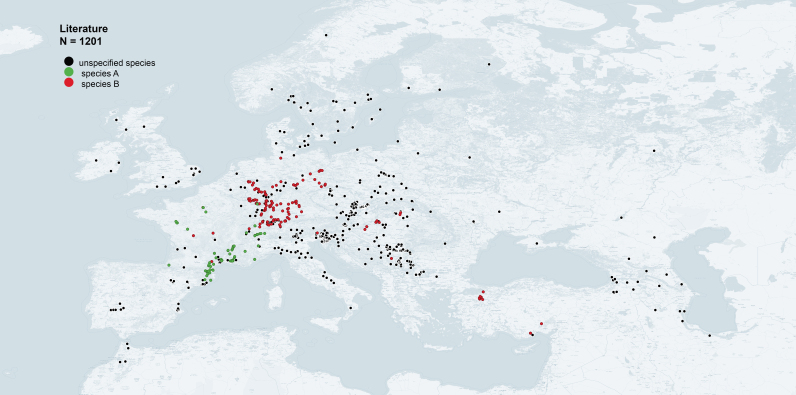
Occurrence data of *Cacopsylla pruni* in the Western Palaearctic, obtained from our literature survey (map generated with QGIS 3.14).

The searches were not restricted by language and were traced back to the first description of *C. pruni* (1763). Each line of the dataset that we make available (see section 'Data resources') corresponds to a reference. For almost all of them, we have retrieved the PDF file of the orignal publication (including old books) which allowed us to verify the information. The corresponding URL is given for each data in the dataset (DOI link or similar link generally giving direct access to the PDF). We systematically tried to specify the locality where the observation was made (see Quality control section). Whenever the information was available, we specified the cryptic species of *C. pruni* (A or B, according to Peccoud et al. 2013) and the collection plant. In the end, we were able to exploit 1201 occurrence data from the literature survey (Fig. 3).

**GBIF data**

**Figure 4.**
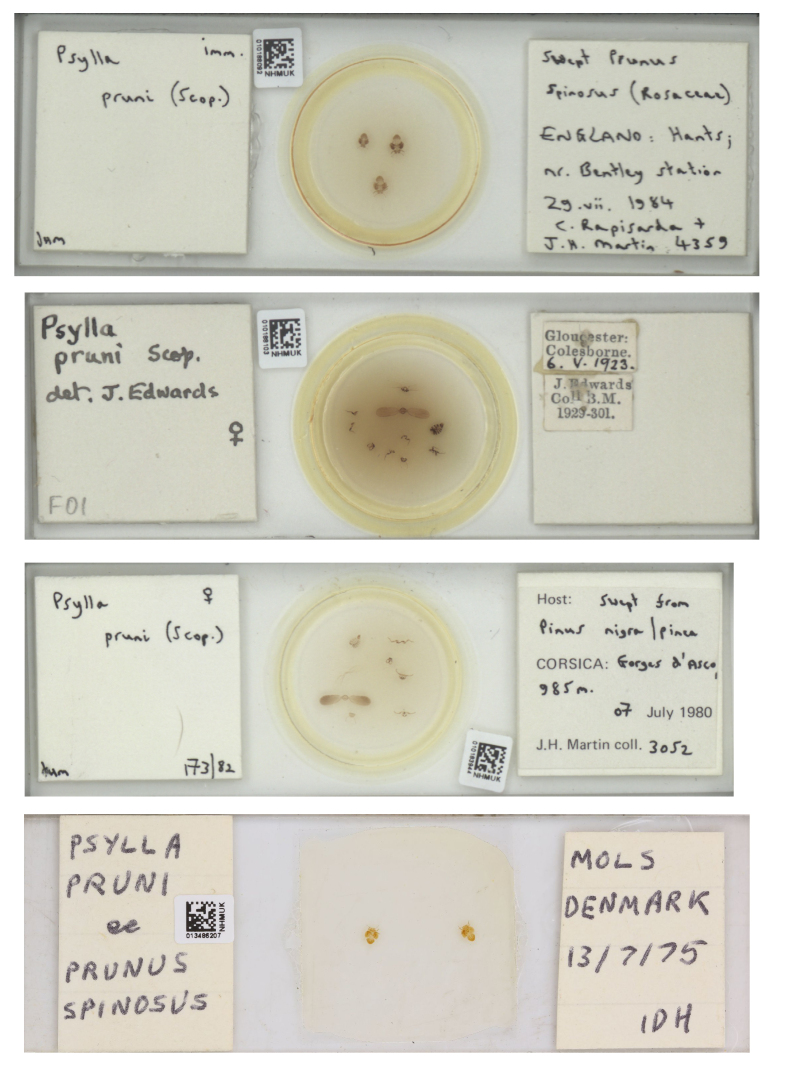
Examples of metadata accessible on the website of the Natural History Museum from links associated with GBIF references (e.g. https://www.gbif.org/occurrence/1265697015).

**Figure 5.**
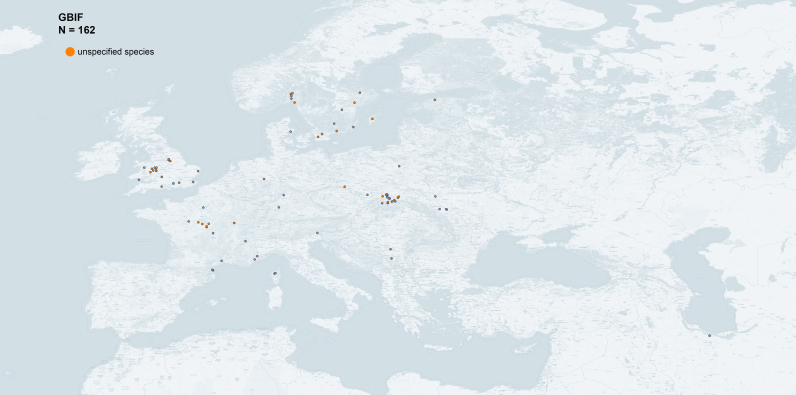
Occurrence data of *Cacopsylla pruni* in Western Palaearctic from the GBIF database (map generated with QGIS 3.14).

A search on the keywords "*Cacopsylla pruni*" returned 245 occurrences in GBIF.org (14 June 2021). The derived dataset with filtered export of GBIF occurrence data is available at this link: https://doi.org/10.15468/dd.rm55g8. Amongst the 245 occurrences, we were able to extract the names of 45 localities with geographic coordinates. For 87 occurrences, for which only the name of the locality was given, we retrieved the geographic coordinates from Google Earth. The database also provided images of scanned slides from the NHM collection (https://www.gbif.org/fr/occurrence/gallery?taxon_key=2012955) from which we retrieved precise information about the sampling (date, location, host plant, collector) (Fig. 4), sometimes redundant with our own information (e.g. data from Iran). Finally, 28 occurrences were derived from information associated with DNA sequences deposited in iBOL (https://ibol.org), including 24 sequences deposited by us and already entered in our dataset (e.g. https://www.ncbi.nlm.nih.gov/nuccore/MH577786). In total, 162 occurrences data have been extracted from GBIF (Fig. 5).

**Sampling data**

For more than 20 years, researchers (Gérard Labonne, Gaël Thébaud, Jean Peccoud, Christian Cocquempot and Nicolas Sauvion) or students of INRAE-Montpellier have collected *C. pruni* individuals. Using a beating tray (80 cm x 80 cm), we collected essentially on *Prunus spinosa* L. (blackthorn) in spring and the rest of the year on *Pinus nigra* J.F Arnold (Black Pine), *Picea abies* (L.) H.Karst. (Common Pitch-fir) and *Abies alba* Mill. (Common Silver Fir). Other congeneric species where sometimes caught, but *C. pruni* individuals were easily recognised by the colour of the fore wing, which is dark brown at the apex and brown in the remaining part. Soon after identification, samples were conserved in 96% ethanol until DNA extraction and then genotyped (for species determination) according to the protocol described by Peccoud et al. (2013).

**Figure 6.**
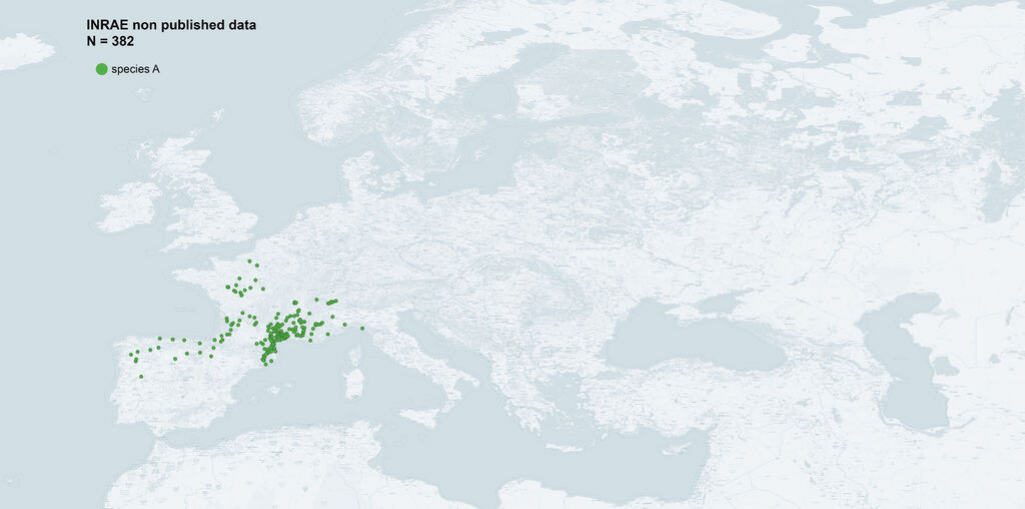
Occurrence data of *s*pecies of *Cacopsylla pruni* A in Western Palaearctic from sampling carried out by INRAE-Montpellier (map generated with QGIS 3.14).

**Figure 7.**
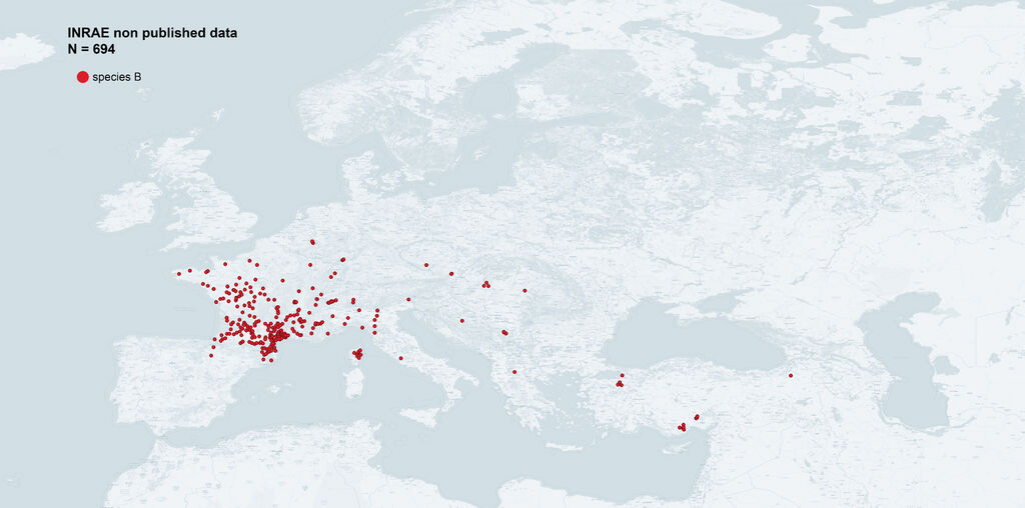
Occurrence data of *s*pecies of *Cacopsylla pruni* B in Western Palaearctic from sampling carried out by INRAE-Montpellier (map generated with QGIS 3.14).

We recorded the GPS coordinates of all collected samples in their wild habitat, geolocalising the bush, hedge or shrub sampled. For the few insects sampled in orchards, we attributed a unique GPS coordinate — corresponding to the centre of each plot — to all the corresponding samples. The name of the locality given in the dataset corresponds to the nearest locality to the sampled point. We sampled mainly France, without restriction to apricot-growing regions and focusing on southern regions where species A and B live in sympatry or in strict allopatry. We also collected samples in Spain, Switzerland and Italy. The addition of these 612 new occurrence data improves the picture of the geographical distribution of the two species, hence it should be valuable for risk assessment, phylogeography or population genetics studies (Fig. 2, Fig. 6, Fig. 7)

### Quality control

We have a strong expertise in the taxonomy of psyllids (Ouvrard 2021). Over the last few years, we have accumulated a large number of references on these insects in an article database, including references that are old and/or difficult to trace. As we had all these articles in PDF or paper or other metadata (e.g. scanned images), we were able to retrieve and thoroughly verify all information concerning *C. pruni* or its synonyms and combinations.

All the specimens that we collected in the field were first carefully visually examined and then genotyped according to Peccoud et al. (2013), which effectively eliminates all risks of misidentification.

Wherever possible, geographic coordinates (in WGS-84 coordinate system) refer to specific localities. We used Google Earth to search and reference each locality name found in literature or GBIF, being careful about homonymy and translation of names and possible changes of country names. We consider the precision of these geographical coordinates to be a few kilometres, as authors rarely give very precise coordinates of their sampling points. Conversely, whenever we found geographical coordinates in GBIF, we plotted them on a Google Earth map to identify the closest locality and to check consistency with other information provided (name of the region, country etc.). When no locality name was given, precision may vary from city to province, region or country (e.g. "USSR: South European Part"). In this case, we specified that the “locality is not stated". For data points only specifying countries, we provided the GPS coordinates of the country centres extracted from Google Earth, for lack of a better option. We, therefore, included a column with the estimated precision for each record, stressing that some of these data should be used with caution depending on the level of precision required for analyses. Conversely, GPS coordinates of our own collected samples (see previous section) have an accuracy of a few metres. Each point was first geolocalised with a portable GPS and then checked on Google Maps.

### Step description

Most field names of the dataset were chosen according to the Darwin Core format (Wieczorek et al. 2012) and the latest version of the list of core terms as of 28-10-2020 (http://rs.tdwg.org/dwc/version/terms/2020-10-28.htm): “catalogNumber”, “phylum”, “order”, “genus”, “acceptedNameUsage”, “Occurrence”, “country”, “countryCode”, “locationRemarks”, “locality”, “coordinateUncertaintyInMetres”, “decimalLatitude”, “decimalLongitude”, “ownerInstitutionCode”, “locationAccordingTo”, “dateIdentified”, “eventDate”, “associatedReferences”. We have added 11 columns with names not defined by Darwin Core: “suborder”, “superfamily”, “family“, “subfamily”, “speciesA”, “speciesB”, “hostPlantFamily”, “hostPlantLatinName”, “hostPlantVernacularName”, “sourceCategory”, “page”.

## Geographic coverage

### Description

The database covers the entire known geographic range of the two cryptic species of the psyllid *C. pruni*, from Morocco to Norway and from Portugal to Mongolia.

We have also extended our search to other countries where either species could potentially be found, in particular countries where different species of *Prunus* are described in wild or cultivated ecological compartments (e.g. Japan, China, USA, Canada) and where these psyllids could be phytoplasma vectors. Whenever possible, we relied on checklists from recognised taxonomists to ensure the veracity of the information before concluding as "absence" (e.g. Inoue 2010, Maw et al. 2000).

### Coordinates

33.815458 and 65.59623333 Latitude; -8.383379 and 112.52588611 Longitude.

## Taxonomic coverage

### Description

The data paper focuses on two cryptic species of Cacopsylla (Thamnopsylla) pruni (Scopoli, 1763), currently referred to as A and B. Species of *Cacopsylla pruni* show clear genetic differences despite being morphologically and ecologically indistinguishable (Peccoud et al. 2013, Peccoud et al. 2018). These psyllids are sternorrhynchans of the order Hemiptera, belonging to the superfamily Psylloidea, family Psyllidae and subfamily Psyllinae according to the classification by Burckhardt et al. (2021).

## Temporal coverage

**Living time period:** 1763-2020.

### Notes

Literature data cover 1763 to 2020.

INRAE data cover 1998 to 2020.

## Usage licence

### Usage licence

Оpen Data Commons Open Database License (ODbL)

## Data resources

### Data package title

Compilation of occurrence data for two psyllid species of the *Cacopsylla pruni* complex (Hemiptera: Psylloidea).

### Resource link

https://doi.org/10.15454/VC9UR5

### Number of data sets

1

### Data set 1.

#### Data set name

Cacopsylla pruni_occurrences_v29.csv

#### Data format

Darwin Core Archive

#### Number of columns

30

#### Character set

text/tab-separated-values

#### Download URL

https://data.inrae.fr/dataset.xhtml?persistentId=doi: 10.15454/VC9UR5

#### Data format version

10

#### 

**Data set 1.** 

Column label	Column description
catalogNumber	An identifier which assigns a unique code to each of the 1975 records (NS0001 to NS1975).
phylum	The full scientific name of the phylum in which the taxon is classified.
class	The full scientific name of the class in which the taxon is classified.
order	The full scientific name of the order in which the taxon is classified.
suborder	The full scientific name of the suborder in which the taxon is classified.
superfamily	The full scientific name of the superfamily in which the taxon is classified.
family	The full scientific name of the family in which the taxon is classified.
subfamily	The full scientific name of the subfamily in which the taxon is classified.
genus	The full scientific name of the genus in which the taxon is classified.
acceptedNameUsage	The full name, with authorship and date information of the currently valid (zoological) taxon.
Occurrence	An existence of an Organism (sensu http://rs.tdwg.org/dwc/terms/Organism) at a particular place at a particular time. Here, five modalities: "insufficient data" (i.e. insufficient information to determine presence or absence); "probable absence" (i.e. no presence data yet found in records); "probable presence" (i.e. presence very likely, but not yet confirmed); "confirmed presence".
speciesA	Information concerning the assignment of the specimens of a population (i.e. caught on the same day in the same locality on the same host plant) to species A of *C. pruni*. Three modalities: "not genotyped"; "not species A" (i.e. no individual of genotype A was found in the population analysed, but individuals of species B); "species A" (i.e. at least one individual of genotype A found in the population analysed). Genotyping was based on Peccoud et al. (2013).
speciesB	Information concerning the assignment of the specimens of a population (i.e. caught on the same day in the same locality on the same host plant) to species B of *C. pruni*. Three modalities: "not genotyped"; "not species B" (i.e. no individual of genotype B was found in the population analysed, but individuals of species A); "species B" (i.e. at least one individual of genotype B found in the population analysed). Genotyping was based on Peccoud et al. (2013).
country	Names of the countries where the individual(s) attributed to *C. pruni* have been recorded, according the universally applicable code ISO 3166-2:2013.
countryCode	Two-letter country codes defined in ISO 3166-1, part of the ISO 3166 standard to represent countries where species have been described.
locationRemarks	Comments or notes about the location.
locality	The specific description of the place. The locality is given as accurately as possible (precise address, village, town), but may sometimes be imprecise (e.g. mountain, region) or even absent (NA="locality not stated"). See column "coordinateUncertaintyInMetres" for more details on uncertainty.
coordinateUncertaintyInMetres	The horizontal distance (in metres) from the given decimalLatitude and decimalLongitude describing the smallest circle containing the whole of the Location. Leave the value empty if the uncertainty is unknown, cannot be estimated or is not applicable (because there are no coordinates). Zero is not a valid value for this term, for example, 30 m = margin of error in the measurement of coordinates using a GPS navigator; 1000 or 10000 m = uncertainty attributed to most locality names in literature, in the absence of more precise information; 50000 m = uncertainty when only the name of the region/province is known.
decimalLatitude	The geographic latitude (in decimal degrees according to the geodetic coordinate reference system EPSG 4326) of the geographic centre of a location. Positive values are north of the Equator, negative values are south of it. Legal values lie between -90 and 90, inclusive.
decimalLongitude	The geographic longitude (in decimal degrees according to the geodetic coordinate reference system EPSG 4326) of the geographic centre of a location. Positive values are east of the Greenwich Meridian, negative values are west of it. Legal values lie between -180 and 180, inclusive.
hostPlantFamily	Six modalities: "Fabaceae"; "Pinaceae"; "Rosaceae"; "Salicaceae"; "unknown" (specimens collected by sweeping or Malaise trap); "unspecified species". Here "host plant" is taken in the broadest sense, i.e. plants on which a psyllid species completes its immature to adult life cycle or shelter plant (plants on which adult psyllids overwinter and on which they may feed) or casual plant (plants on which adult psyllids land, but do not feed).
hostPlantLatinName	Latin name of the host plant species (i.e. host plant sensu stricto, shelter plant or casual plant) according to the International Code of Nomenclature for algae, fungi and plants (https://www.iaptglobal.org/). For example, *Picea abies* (L.) H.Karst., *Prunus spinosa* L. etc.
hostPlantVernacularName	Vernacular English name of the host plant species.
sourceCategory	The three different sources of information used to compile the dataset: "GBIF" (i.e. data from the Global Biodiversity Information Facility); "literature" (i.e. any data resulting from a text-mining from different sources - manuscript, book, article etc. - accessible or not on the web); "INRAE" (i.e. data from collections by INRAE Montpellier, not published to date).
ownerInstitutionCode	The name (or acronym) in use by the institution having ownership of the object(s) or information referred to in the record.
locationAccordingTo	Information about the source of this Location information. Could be a publication (gazetteer), institution or team of individuals. Here, detailed title of the original reference associated with the locality; "no data" (i.e. no information found for a particular country, for example, Kyrgyzstan, Malta).
dateIdentified	The date on which the subject was determined as representing the Taxon. Here, year of publication of the reference cited in the "locationAccordingTo" column.
page	Page where the original information about the locality can be found in the reference cited in the "locationAccordingTo" column.
eventDate	The date-time or interval during which an Event occurred. For occurrences, this is the date-time when the event was recorded. Here, year(s) or date of sampling or observation in the locality according to the information in the "locationAccordingTo" column.'1996' (some time in the year 1996). '2010-06' (some time in June 2010). '2010-02-12' (some time during 12 February 2010). '2007/2010' (some time during the interval between the beginning of the year 2007 and the end of the year 2010).
associatedReferences	A list (concatenated and separated) of identifiers (publication, bibliographic reference, global unique identifier, URI) of literature associated with the occurrence. Here, URL by which the original information can be retrieved (downloadable PDF file in open access, link to the publisher of a non-open access reference, direct link to the original GBIF occurrence etc.).
